# Errors in the Results

**DOI:** 10.1001/jamanetworkopen.2024.12524

**Published:** 2024-04-16

In the Original Investigation titled “Medical Spending Among US Households With Children With a Mental Health Condition Between 2017 and 2021,”^1^ published March 11, 2024, there was a typo in a percentage in the Results section regarding the increase in pediatric mental health care costs from 2017 to 2021. The original percentage stated it was a 31.1% increase, but it was a 45.2% increase. This article has been corrected.^1^
